# Surveillance for rat hepatitis E in wastewater networks, Italy

**DOI:** 10.1128/spectrum.02675-23

**Published:** 2023-10-18

**Authors:** Andrea Palombieri, Federica Di Profio, Vittorio Sarchese, Paola Fruci, Elisabetta Suffredini, Vito Martella, Carolina Veneri, Giusy Bonanno Ferraro, Pamela Mancini, Giuseppina La Rosa, Barbara Di Martino

**Affiliations:** 1 Department of Veterinary Sciences, University of Teramo, Località Piano d'Accio, Teramo, Italy; 2 Department of Food Safety, Nutrition and Veterinary Public Health, Istituto Superiore di Sanità, Viale Regina Elena, Rome, Italy; 3 Department of Veterinary Medicine, University of Bari Aldo Moro, Valenzano, Italy; 4 Department of Environment and Health, Istituto Superiore di Sanità, Rome, Italy; City University of Hong Kong, Kowloon, Hong Kong, China

**Keywords:** viruses, zoonoses, rat hepatitis E virus, wastewater surveillance, Italy

## Abstract

Rat hepevirus is an emerging zoonotic pathogen associated with human hepatitis and distantly related to the hepatitis E virus. By using broadly reactive primers for hepevirus, we detected viral RNA in 68/155 (43.9%) of sewage specimens collected from 14 wastewater treatment plants serving about half of the population of Abruzzo, Italy. On sequence analysis, the 68 strains showed 79.0%–91.6% identities to reference sequences of rodent and human origin, segregating in different genetic groups with a clear geographic/wastewater treatment plant (WWTP)-related pattern.

**IMPORTANCE:**

Hepatitis E virus (HEV) infection constitutes a significant health problem worldwide. In recent years, in addition to the zoonotic HEV3 and HEV4, emerging highly divergent hepevirus of rat origin (rat HEV [RHEV]) has been associated with human acute and chronic hepatitis. As environmental surveillance can be a complementary tool to explore emerging viruses of human and rodent origin, we investigated the epidemiology and the genetic variability of RHEV targeting 14 wastewater treatment plants in an Italian geographic area considered a hot spot for HEV infection in humans. Our results revealed that RHEV is a significant component of the wastewater microbiota with viral RNA detected in 43.9% of the specimens tested, adding further evidence to the need to investigate more in depth the real burden of RHEV infections in humans.

## OBSERVATION

Hepatitis E virus (HEV) is a leading cause of acute hepatitis worldwide, with an estimated 20 million new infections and over 44,000 reported deaths yearly (https://www.who.int/news-room/fact-sheets/detail/hepatitis-e). HEV is a member of the family *Hepeviridae*, subfamily *Orthohepevirinae* (all mammalian and avian HEVs) (1), and has a linear positive-sense single-stranded RNA ~7.2 kb in length, surrounded by a spherical quasi-enveloped or non-enveloped virion with a diameter of approximately 28–35 nm. The *Orthohepevirinae* subfamily is divided into four genera, with members of the genera *Paslahepevirus, Chirohepevirus,* and *Rocahepevirus* infecting humans and domestic and wild mammals (2). Four major genotypes (HEV1–4), belonging to the species *Paslahepevirus balayani,* have been implicated in human disease with HEV3 and HEV4 infections mainly acquired through zoonotic pathways. In recent years, genetically highly divergent (<52% nucleotide identity) hepeviruses of rat origin, classified in the species *Rocahepevirus ratti* genotype C1 (rat HEV [RHEV]), have also been detected in patients with acute or chronic hepatitis (2 – 7). Thus far, 21 cases of human infections with RHEV have been reported, including 16 in Hong Kong (2 – 4), one in Canada (5), three in Spain (6), and one in France (7), and RHEV is currently considered an emerging cause of hepatitis infection. Since antigenic cross-reactivity has been demonstrated between RHEV antigens and HEV-derived antibodies (6) and specific molecular assays for RHEV diagnostics are currently not available, it is possible that the role of RHEV in human hepatitis has been underlooked. To date, only monoplex in-house–developed and validated reverse transcription (RT) PCRs (2 – 4), quantitative RT-PCR (3, 4), broad-spectrum PCR assay that detects largely divergent HEV variants (5, 6), or metagenomics test based on next-generation sequencing (7) were used in the clinical studies that described the first cases of RHEV infections in humans.

Sewage surveillance has been recognized as a powerful tool to gather information on the epidemiology of infectious diseases in the served population. Untreated wastewater collects viruses excreted by both humans and synanthropic animals, including rodents, thereby providing a comprehensive overview of the viral strains circulating. Herein, to investigate the epidemiology and the genetic variability of RHEV in Italy, we targeted wastewater treatment plants (WWTPs) of Abruzzo region (southern Italy). This geographic area is considered a hot spot for HEV infection in Italy (8).

**Fig 1:**
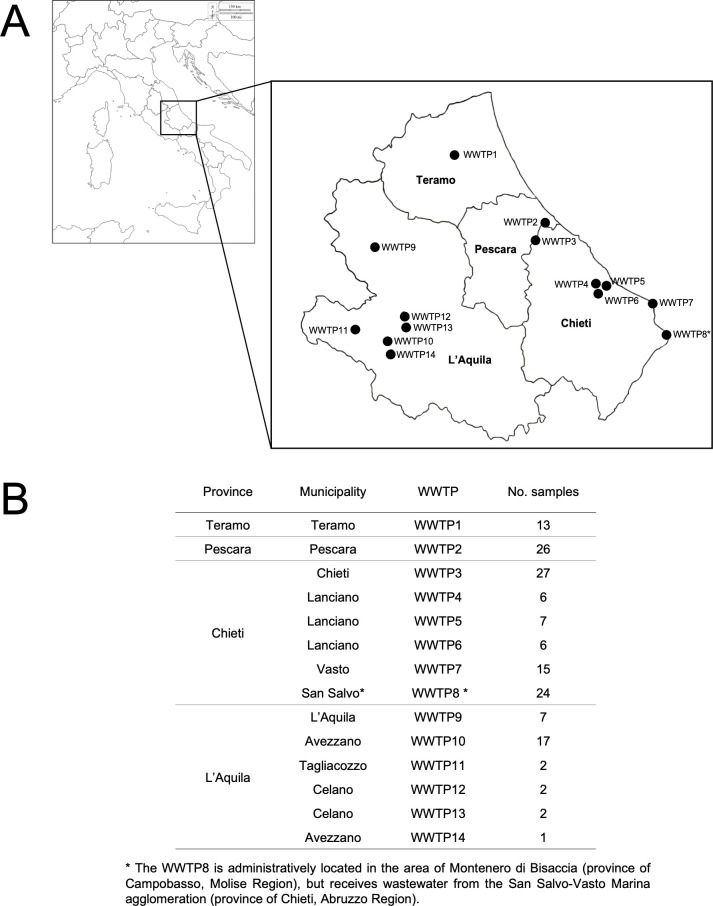
Representation of the geographic map showing the wastewater sampling sites selected in this study from municipal wastewater treatment plants (WWTPs) in Abruzzo (Italy) (**A**) and list of additional attribute information including the municipality and the number of samples collected (**B**). The Abruzzo map was created using Quantum GIS (QGIS) software version 3.22; the Italy map came from d-maps.com (https://d-maps.com/carte.php?num_car=4831&lang=en).

Fourteen WWTPs, serving a total of 538,540 inhabitants (~50.0% of total residents of the region), were selected for the investigation. These WWTPs were evenly distributed across the region, encompassing the four provinces of Abruzzo: L’Aquila, Teramo, Pescara, and Chieti. For most of the targeted sites, wastewater samples (*N* = 155) were collected monthly for approximately 30 mo (between October 2019 and March 2022). The locations and the characteristics of the WWTPs are shown in Fig. 1. Sample concentration took place using viral precipitation with polyethylene glycol 8000 and NaCl (9). Viral RNA was extracted using the NucliSENS magnetic extraction reagents (bioMerieux, Marcy l’Etoile, France) according to the manufacturer’s instructions. The eluted RNA (100 µL) was purified using the OneStep PCR Inhibitor Removal Kit (Zymo Research, Irvine, CA, USA) and stored at −80°C until molecular analysis. We used a pan-hepevirus heminested RT-PCR based on a touchdown protocol and broadly reactive primers designed to amplify a conserved 338-nucleotide (nt) region of the polymerase gene (10), corresponding to nt 4,076–4,430 of the reference strain of the species *Rocahepevirus ratti* (GU345042). Amplicons of the expected size were detected in 75 raw sewage specimens (48.4%, 95% confidence interval (CI) 40.5%–56.3%). The amplicons were purified using a Montage PCRm96 microwell filter plate (Millipore, Billerica, MA, USA) and subjected to Sanger sequencing. Of the 75 positive samples, 7 (9.3%) were characterized as HEV3, while 68 sequences (90.7%) were characterized into the species *Rocahepevirus ratti*, genotype C1. RHEV RNA was detected in 9 out of the 14 WWTPs included in the study, with a prevalence of 65.4% (95% CI 47.1%–83.7%; 17/26) in Pescara, 53.8% (95% CI 26.7%–80.9%; 7/13) in Teramo, 44.7% (95% CI 34.1%–55.3%; 38/85) in Chieti, and 19.3% (95% CI 0.5%–33.2%; 6/31) in L’Aquila and with an overall rate of 43.9% (95% CI 36.1%–51.7%; 68/155). The virus was recovered throughout the entire collection period in all four provinces, with detection rates from 37.2% to 66.7% (Table 1).

**TABLE 1:** Molecular prevalences and temporal distribution of positive samples for each WWTP in the provinces of Teramo, Pescara, Chieti, and L’Aquila (Abruzzo region, Italy)

WWTP^*a*^	Municipality	Province	Positive samples	Sampling years			
				2019	2020	2021	2022
WWTP1 Villa Pavone	Teramo	Teramo	7/13 (53.8%)	0	0	5/10 (50.0%)	2/3 (66.7%)
		Teramo—total	7/13 (53.8%)	0	0	5/10 (50.0%)	2/3 (66.7%)
WWTP2 Via Raiale	Pescara	Pescara	17/26 (65.4%)	1/1 (100%)	7/11 (63.6%)	8/12 (66.7%)	1/2 (50.0%)
		Pescara—total	17/26 (65.4%)	1/1 (100%)	7/11 (63.6%)	8/12 (66.7%)	1/2 (50.0%)
WWTP3 San Martino	Chieti	Chieti	6/27 (22.2%)	1/2 (50.0%)	2/11 (18.2%)	2/12 (16.7%)	1/2 (50.0%)
WWTP4 S. Liberata	Lanciano	Chieti	1/6 (16.7%)	0	0	1/6 (16.7%)	0
WWTP5 Santa Croce	Lanciano	Chieti	4/7 (57.1%)	0	0	4/7 (57.1%)	0
WWTP6 Villa Martelli	Lanciano	Chieti	2/6 (33.3%)	0	0	2/6 (33.3%)	0
WWTP7 Punta Penna	Vasto	Chieti	10/15 (66.7%)	2/2 (100%)	1/2 (50.0%)	6/10 (60.0%)	1/1 (100%)
WWTP8 Padula	San Salvo	Campobasso	15/24 (62.5%)	2/4 (50.0%)	4/4 (100%)	8/15 (53.3%)	1/1 (100%)
		Chieti—total	38/85 (44.7%)	5/8 (62.5%)	7/17 (41.2%)	23/56 (41.1%)	3/4 (75.0%)
WWTP9 Pile	L’Aquila	L’Aquila	0/7	0	0	0/7	0
WWTP10 Puzzillo	Avezzano	L’Aquila	6/17 (35.3%)	0/1	4/6 (66.7%)	2/10 (20.0%)	0
WWTP11 Tagliacozzo	Tagliacozzo	L’Aquila	0/2	0	0	0/2	0
WWTP12 Rio Pago	Celano	L’Aquila	0/2	0	0	0/2	0
WWTP13 Rio La Foce	Celano	L’Aquila	0/2	0	0	0/2	0
WWTP14 Borgo Via Nuova	Avezzano	L’Aquila	0/1	0	0	0/1	0
		L’Aquila—total	6/31 (19.3%)	0/1	4/6 (66.7%)	2/24 (8.3%)	0
		Total	68/155 (43.9%)	6/10 (60.0%)	18/34 (52.9%)	38/102 (37.2%)	6/9 (66.7%)

^*a*^ WWTP: wastewater treatment plant.

**Fig 2:**
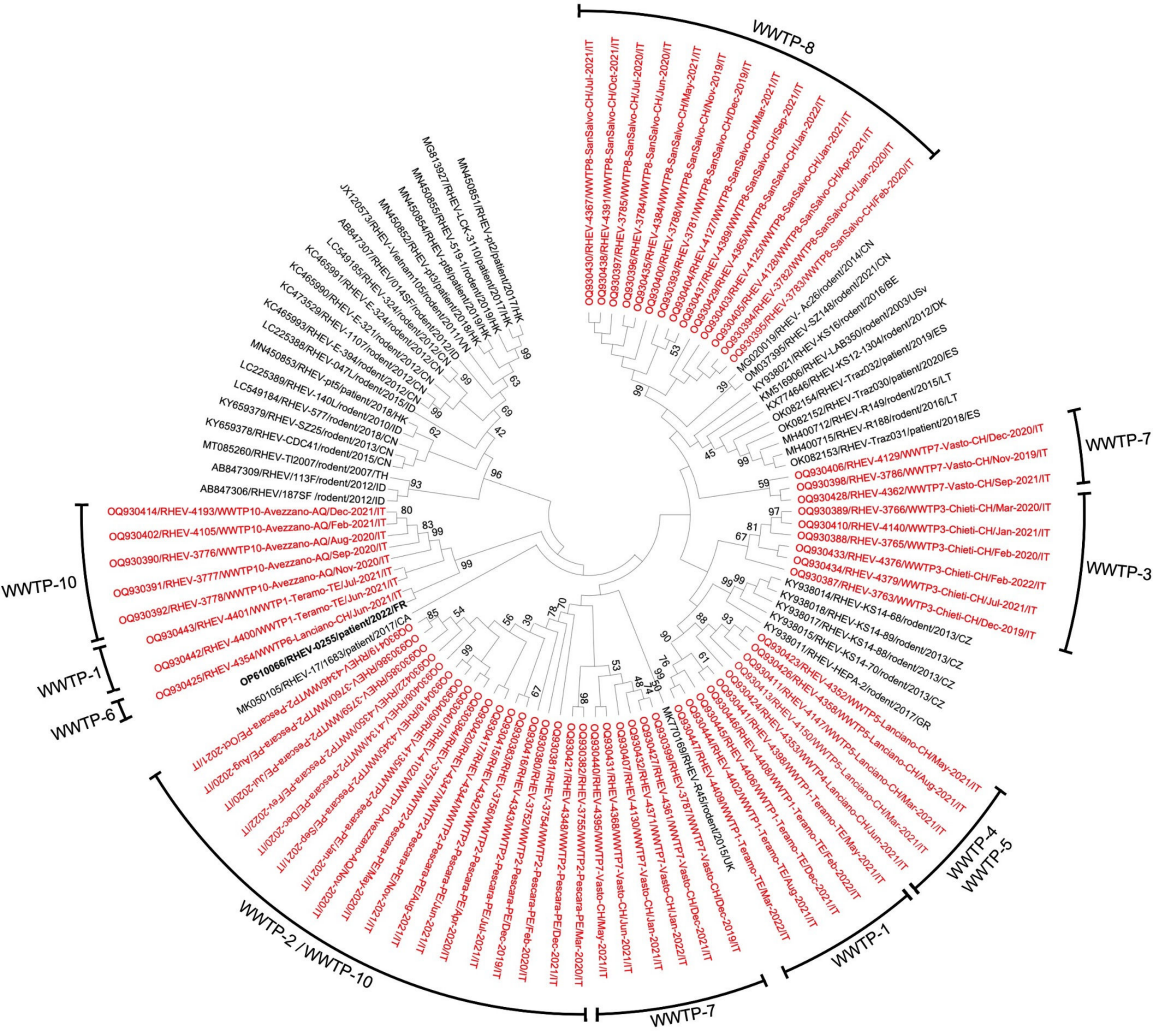
The evolutionary history was inferred using the neighbor-joining method. The percentage of replicate trees in which the associated taxa clustered together in the bootstrap test (1,000 replicates) are shown next to the branches. The evolutionary distances were computed using the maximum composite likelihood method and are in the units of the number of base substitutions per site. This analysis involved a total of 104 nucleotide sequences. The sequences detected in this study are in red.

The RHEV sequences obtained in this study (GenBank accession numbers OQ930380–OQ930447) shared 82.5%–95.8% nt identity to each other, while identities to all the sequences of rodent and human origin to date available in GenBank were 79.0%–91.6%. On phylogenetic analysis (Fig. 2), the Italian RHEV strains grouped in different genetic clusters with a clear geographic/WWTP-related pattern, a finding that would be consistent with the persistence of the same strain within local rat populations. Interestingly, the sequence detected in WWTP-6 (Lanciano, Chieti) formed a well-separated subclade (bootstrap value of 86%) with the human RHEV strain recently identified in France (7) from the paraffin-embedded liver biopsy of an immunosuppressed kidney-transplanted patient with cirrhosis.

All the 68 samples containing the sequence of RHEV were screened using a quantitative RT-PCR (qRT-PCR) specific for HEV, targeting a highly conserved region at the ORF2/ORF3 overlap, and able to detect the four major HEV genotypes (e.g., *Paslahepevirus balayani*) (11). However, the qRT-PCR did not detect RHEV RNA, ruling out the presence of mixed infections in the samples and confirming the specificity of the real-time HEV assay (2, 6).

Our data revealed that RHEV is a significant component of the wastewater microbiota. This could be accounted for by direct fecal contamination of untreated water by the rodents inhabiting the sewage system. Rodent-associated hepeviruses were first identified in 2010 in fecal and liver specimens from wild rats in Germany (12). Since then, RHEV has been repeatedly detected in several countries in Europe, Asia, and USA, demonstrating a wide distribution in commensal rat populations (*Rattus norvegicus*, *Rattus rattus*, and others) (13). Rat species are now recognized as the main host animals of RHEV, and for this reason, it has been hypothesized that close contact with rats or with rat fecal droppings could be the primary transmission route for human infection. Nevertheless, out of the 21 cases of infection worldwide reported thus far (2 – 7), this possibility has been deemed plausible only for one patient living in Hong Kong, in a housing estate with evidence of rat infestation in the refuse bins outside his home, while for the other 20 patients, the source of infection remained undetermined. Accordingly, alternative transmission dynamics should be considered. RHEV sequences were recently detected in effluent wastewater at the Rya treatment plant in Gothenburg (Sweden) by NGS (14). The occurrence of RHEV in urban wastewater raises the question as to whether contaminated surface waters can be responsible for waterborne transmission. In Italy, HEV3 RNA has been detected in river waters receiving wastewater discharges and from shellfish, marine waters, and underwater sewage discharges (15, 16). Further investigations integrating environmental/animal/food surveillance are pivotal to provide a clearer picture of the pathways of RHEV infection. Also, due to the inability of commonly used diagnostic tools to detect RHEV, the global prevalence of infection in the human population is still unknown. Therefore, it will be important to validate specific molecular techniques for RHEV and to include RHEV in the diagnostic algorithm of human acute hepatitis.

## Data Availability

The 68 nucleotide sequences generated in this study were deposited in GenBank under accession numbers OQ930380–OQ930447.
